# Live imaging and functional changes of the inner ear in an animal model of Meniere’s disease

**DOI:** 10.1038/s41598-020-68352-0

**Published:** 2020-07-23

**Authors:** Akinobu Kakigi, Naoya Egami, Natsumi Uehara, Takeshi Fujita, Ken-ichi Nibu, Shinji Yamashita, Tatsuya Yamasoba

**Affiliations:** 1 Department of Otolaryngology-Head and Neck Surgery, Kobe University, Graduate School of Medicine, 7-5-1 Kusunoki-cho, Chuo-ku, Kobe, Hyogo 650-0017 Japan; 2 Department of Otolaryngology, Faculty of Medicine, The University of Tokyo, Tokyo, 113-8655 Japan; 3 Research Center for Advanced Science and Technology, The University of Tokyo, Tokyo, 153-8904 Japan

**Keywords:** Neuroscience, Auditory system, Diseases

## Abstract

The symptoms of Meniere’s disease (MD) are generally considered to be related to endolymphatic hydrops (EH). There are many recent reports supporting the possibility that vasopressin (VP) is closely linked to the formation of EH in Meniere’s disease. Based on this, we developed a clinically relevant animal model of Meniere’s disease in which a VP type 2 receptor agonist was administered after electrocauterization of the endolymphatic sac. We report live imaging of the internal structure, and functional changes of the inner ear after electrocauterization of the endolymphatic sac and administration of a VP type 2 receptor agonist. In this model, the development of EH was visualized in vivo using optical coherence tomography, there was no rupture of Reissner’s membrane, and low-tone hearing loss and vertiginous attacks were observed. This study suggested that acute attacks are caused by the abrupt development of EH. This is the first report of live imaging of the development of EH induced by the administration of a VP type 2 receptor agonist.

## Introduction

Meniere’s disease (MD) is a well-known inner ear disorder characterized by several symptoms, including recurring attacks of vertigo typically lasting for hours, fluctuating sensorineural hearing loss, and tinnitus. In the early stage of MD, hearing loss usually involves the low frequencies. MD is histologically characterized by endolymphatic hydrops (EH) in the inner ear^1,2^. There is considerable evidence that water homeostasis in the inner ear is partly regulated via the vasopressin-aquaporin 2 (VP-AQP2) system^3–13^ as follows: (1) plasma levels of arginine VP are higher in patients with MD and may depend on the phase that the patient is in^3,5,6^, (2) acute and chronic application of arginine VP produces EH in guinea pigs and rats^4,7,10^, (3) V2 receptor mRNA is expressed in the rat and human inner ear^8,11–13^, and (4) expression of V2 receptor mRNA in the rat inner ear is down-regulated by VP application^9^. Since the discovery of aquaporin (AQP) water channels^14^, precise regulation of water reabsorption was proposed to largely depend on the regulation of AQP2 channels and water permeability may there fore change rapidly in response to vasopressin (VP) in the kidney^15^. Such evidence led to the assumption that the production of endolymph is controlled by the VP-AQP2 system in the inner ear. If this is the case, EH, a morphological characteristic of MD, reflects the misregulation of the VP-AQP2 system in inner ear fluid. Based on human and experimental studies, we recently developed a more suitable animal model of MD^16^. This model consists of the combination of endolymphatic sac dysfunction and administration of a vasopressin type 2 receptor agonist (desmopressin). Although episodes of imbalance were rarely observed in previous models of EH, vertigo was observed in our new animal model. Moreover, we recently visualized the internal structure of the inner ear using optical coherence tomography (OCT) in vitro^17^. OCT uses low-coherence interferometry to produce a two-dimensional image of internal tissue microstructures^18^. It uses light to discern intrinsic differences in tissue structure and coherence gating to localize the origin of the reflected optic signal. Internal tissue microstructures can be visualized with axial and lateral spatial resolutions on the order of 10 µm, and a depth of penetration of approximately 2–3 mm depending on tissue translucency. This technology has become widely established for clinical application in the fields of ophthalmology and dermatology to visualize the translucent tissues of the eye^19^ and superficial tissues of the skin^20^. Although visualization of the internal structures of the inner ear has been employed to identify certain pathological conditions, the resolution and quality were reported to be unsatisfactory, mainly due to the thick bony capsule surrounding this endorgan^21^.

In the current study, we investigated the inner ear function, and observed the internal structure using OCT in vivo after electrocauterization of the endolymphatic sac and administration of VP type 2 receptor agonist. Moreover, the development of EH was clearly visualized in vivo. During EH development, there was no rupture of Reissner’s membrane, but low-tone hearing loss and vertiginous attacks were observed. This suggests that acute attacks of MD are caused by the abrupt distention of Reissner’s membrane. This is the first report of live imaging of the development of EH during Meniere’s disease attacks. This study was approved by the Tokyo University Animal Care and Use Committee.

## Results

### In vivo OCT imaging

**Figure 1:**
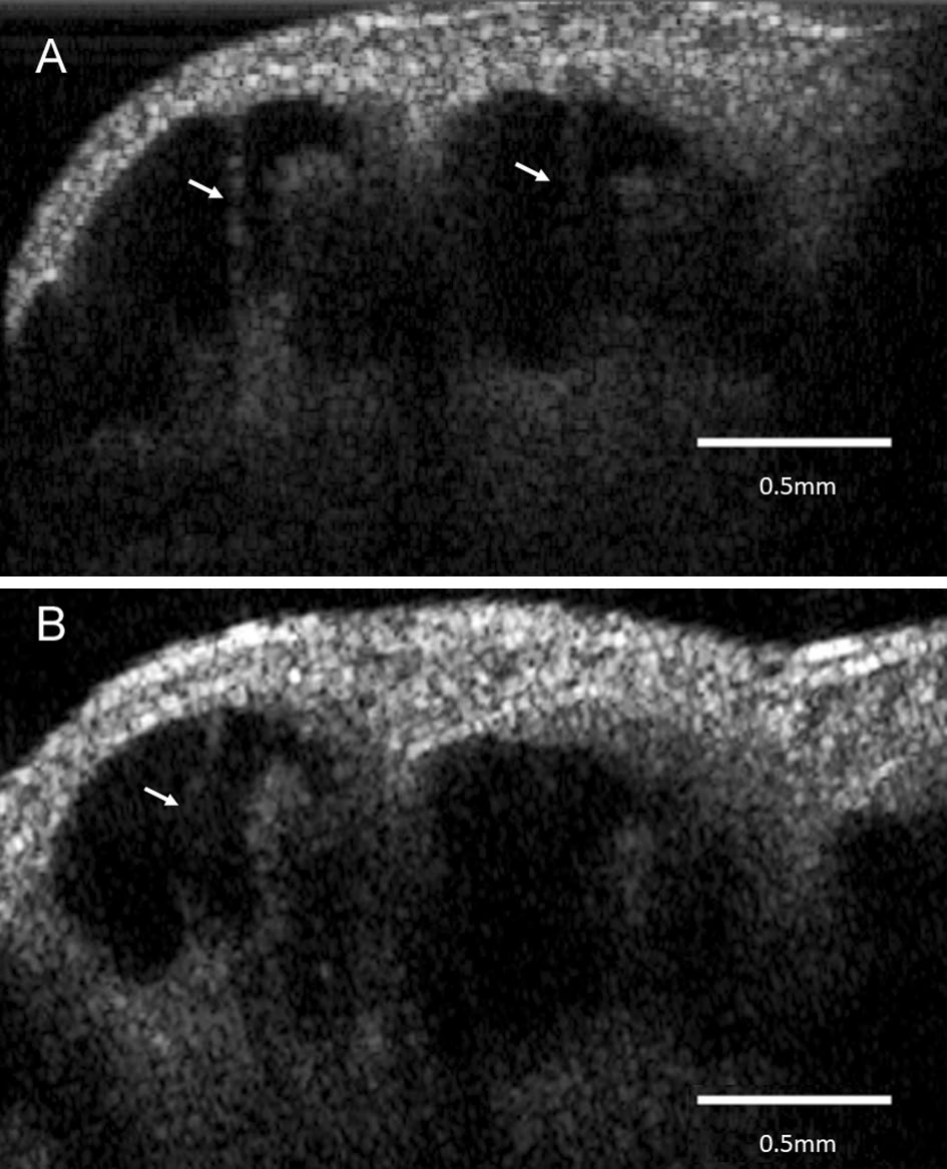
Two-dimensional OCT cross-sectional image of the normal cochlea in vivo. (**A**) The otic capsule, modiolus, Reissner’s membrane (arrow), organ of Corti, spiral limbs, and lateral wall consisting of the stria vascularis and spiral ligament in the apical and third turns of the mid-modiolar section were observed in a 4-week-old animal. The scala tympani, media, and vestibule were clearly distinguishable. (**B**) As the translucency of the bony wall decreases after 6 weeks of age in normal guinea pigs, Reissner’s membrane of the third turn was hardly visualized in an 8-week-old animal.

**Figure 2:**
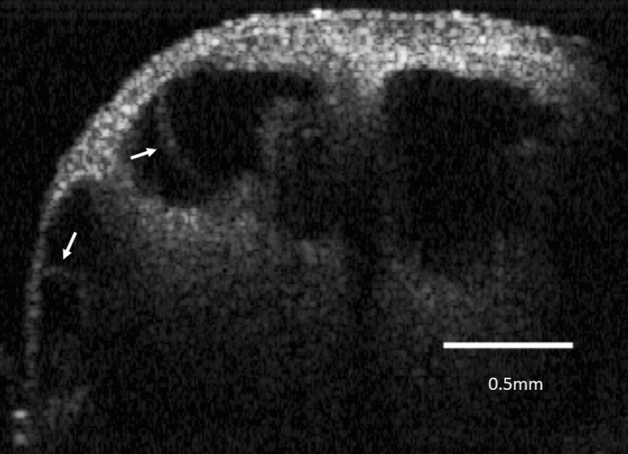
Two-dimensional OCT cross-sectional image of the endolymphatic hydrops in vivo. Representative cross-sectional OCT image of the cochlea in an animal that underwent electrocauterization of the ES. Distinct hydrops was noted in the apical turn. The spiral ligament, stria vascularis, and organ of Corti were normal. Arrow = distention of Reissner’s membrane.

**Figure 3:**
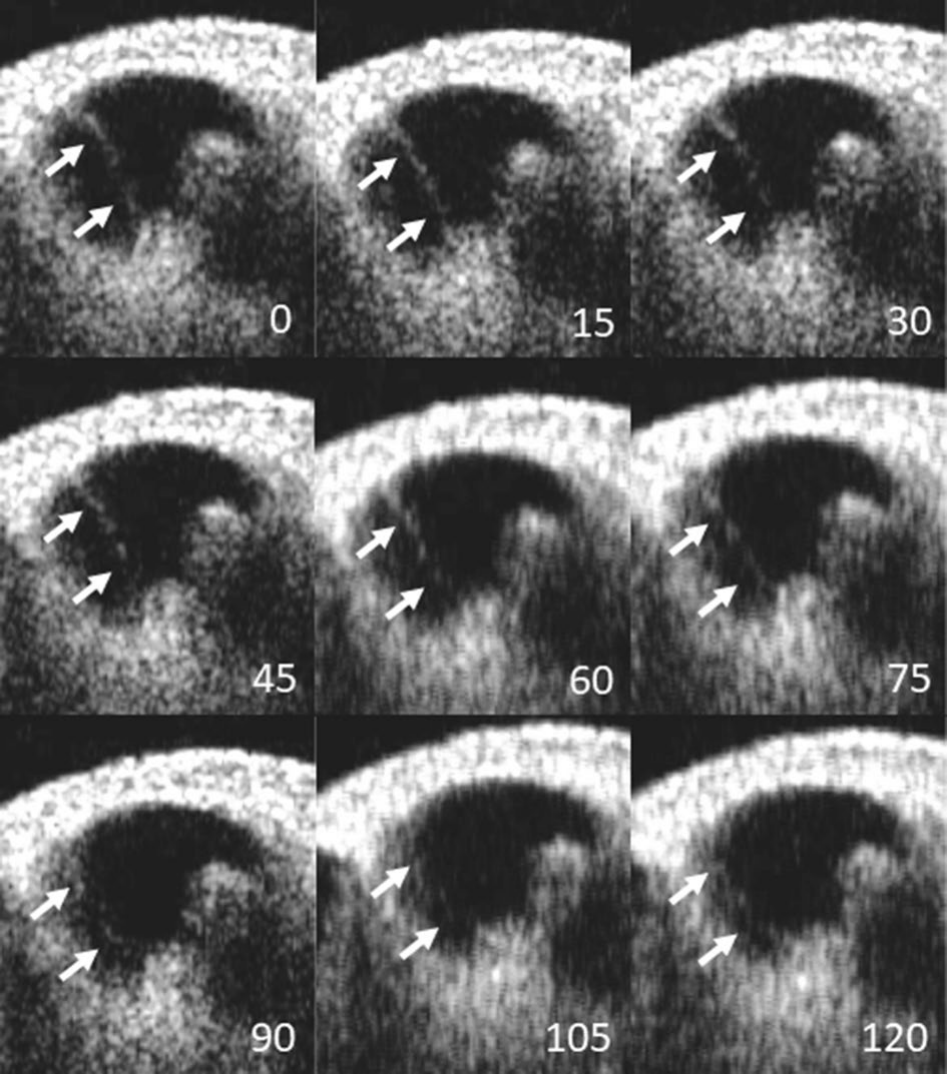
Time course of the development of endolymphatic hydrops after the administration of desmopressin 1 week after electrocauterization of the ES. Endolymphatic hydrops developed after the administration of desmopressin. Numbers indicate the time in minutes after the administration of desmopressin. Arrow = distention of Reissner’s membrane.

**Figure 4:**
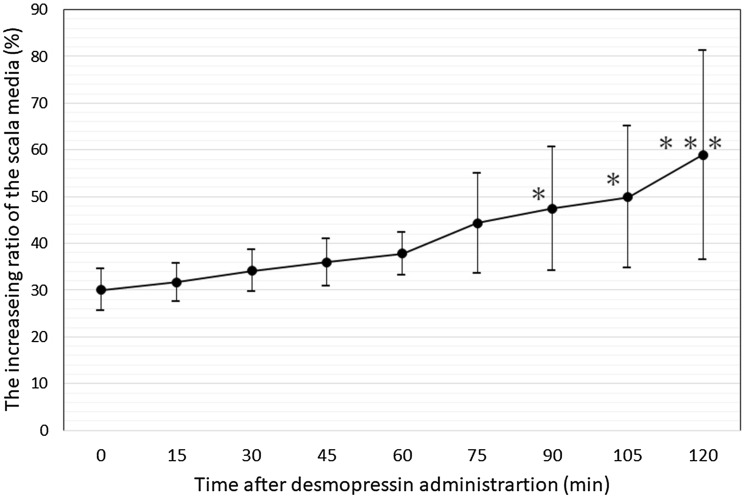
Time course of the increase in the cross-sectional area of the scala media after the administration of desmopressin 1 week after electrocauterization of the ES. Data are presented as the mean ± SD. Data were compared by Tukey’s test and changes were regarded as significant when *P* < 0.05. *P < 0.05; ***P < 0.001.

The guinea pig cochlea OCT images were obtained through a surgically opened bulla. The in vivo 2-D OCT cross-sectional image of the normal cochlea of a 4-week-old animal through the intact osseous otic capsule is shown in Fig. 1. We were able to identify not only the otic capsule and modiolus, but also Reissner’s membrane, the organ of Corti, the spiral limbs, and the lateral wall consisting of the stria vascularis and spiral ligament in the apical and third turns of the mid-modiolar section (Fig. 1A). The scala tympani, media, and vestibule were clearly distinguishable. The translucency of the bony wall gradually decreases with age in normal guinea pigs. As presented in our previous report^17^, whole cochlear images were obtained by in vitro OCT imaging (Supplementary Fig. 1 in Supplementary Materials). However, Reissner’s membrane of the third turn was hardly visualized in 6-week-old animals by in vivo OCT imaging (Fig. 1B). OCT demonstrated the presence of EH 4 weeks after electrocauterization of the ES, which was consistent with the report by Kimura and Schuknecht stating that obliteration of the ES leads to EH^22^. They also noted vestibular disturbance to some degree, but we did not observe such findings. As shown in Fig. 2, distinct hydrops was observed in the apical turn. The spiral ligament, stria vascularis, and organ of Corti appeared normal. As this animal was 8 weeks old, the translucence of the bony wall was lower due to aging and Reissner’s membrane of the apical turn was only visualized. The increase (%) in the cross-sectional area of the scala media (increasing ratio: IR) in the apical turn among the normal, hydrops, and hydrops with desmopressin groups is shown in Table 1. The IRs of the hydrops and hydrops with desmopressin groups significantly increased compared with that of the normal group. As in our previous report^16^, histopathological examination demonstrated the presence of EH in all turns in the hydrops and hydrops with desmopressin groups. Thus, we consider the findings obtained from the apical turn to represent those of the entire cochlea. The time-course of the development of EH after the administration of desmopressin 1 week after electrocauterization of the ES is shown in Fig. 3. The numbers in Fig. 3, indicate the time in minutes after the administration of desmopressin. Slight distention of Reissner’s membrane before the administration of desmopressin is shown in Fig. 3. EH developed after the administration of desmopressin (Movie 1 in Supplementary Materials). There was no rupture of Reissner’s membrane. The time course of IR change after the administration of desmopressin is shown in Fig. 4. From 90 min after administration, the IR significantly increased.

**Table 1:** The increase in the cross-sectional area of the scala media.

	Normal	Hydrops	Hydrops with desmopressin
IR %	19.0 ± 5.4	50.3 ± 17.8*	58.9 ± 22.3*
n	5	5	5

Data are presented as the mean ± SD. Data were compared by Tukey’s test and changes were regarded as significant when *P* < 0.05. *P < 0.05.

There was no significant difference in IR between the hydrops and hydrops with desmopressin groups. However, EH was thought to develop gradually over 4 weeks in the hydrops group but rapidly in the hydrops with desmopressin group. In our animal model, this is the most different point from that reported by Kimura and Schuknecht^22^.

### Vestibular function

**Figure 5:**
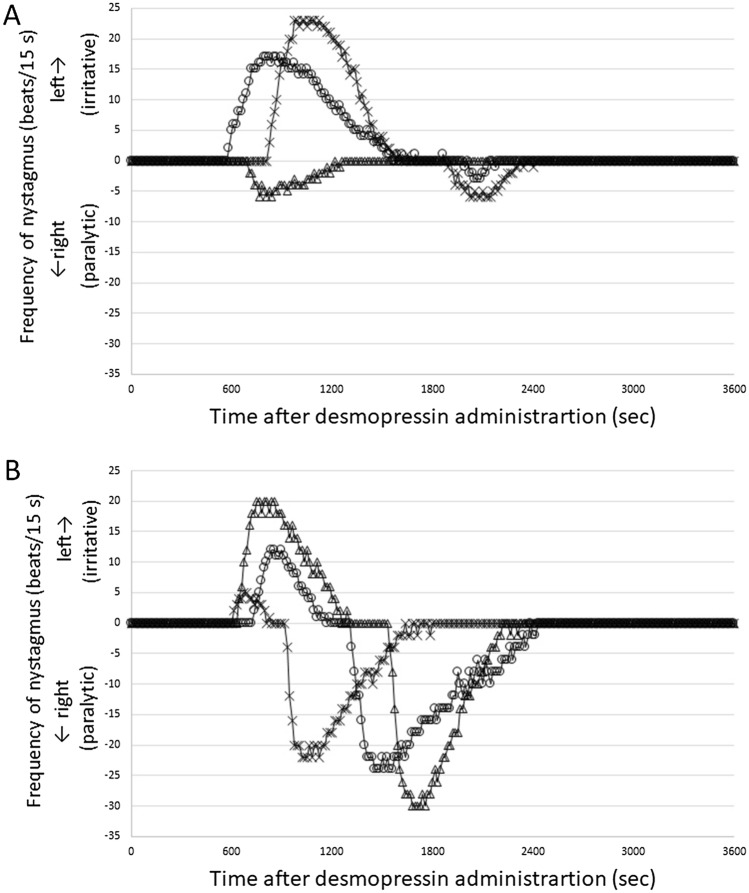
Time course of spontaneous nystagmus after desmopressin administration. (**A**) Animals administered desmopressin 1 week after electrocauterization of the ES (n = 3). (**B**) Animals administered desmopressin 4 weeks after electrocauterization of the ES (n = 3). Positive and negative values of the frequency of nystagmus indicate nystagmus toward the left (surgical side) and right, which are irritative and paralytic nystagmus, respectively. Each symbol represents 1 animal.

**Figure 6:**
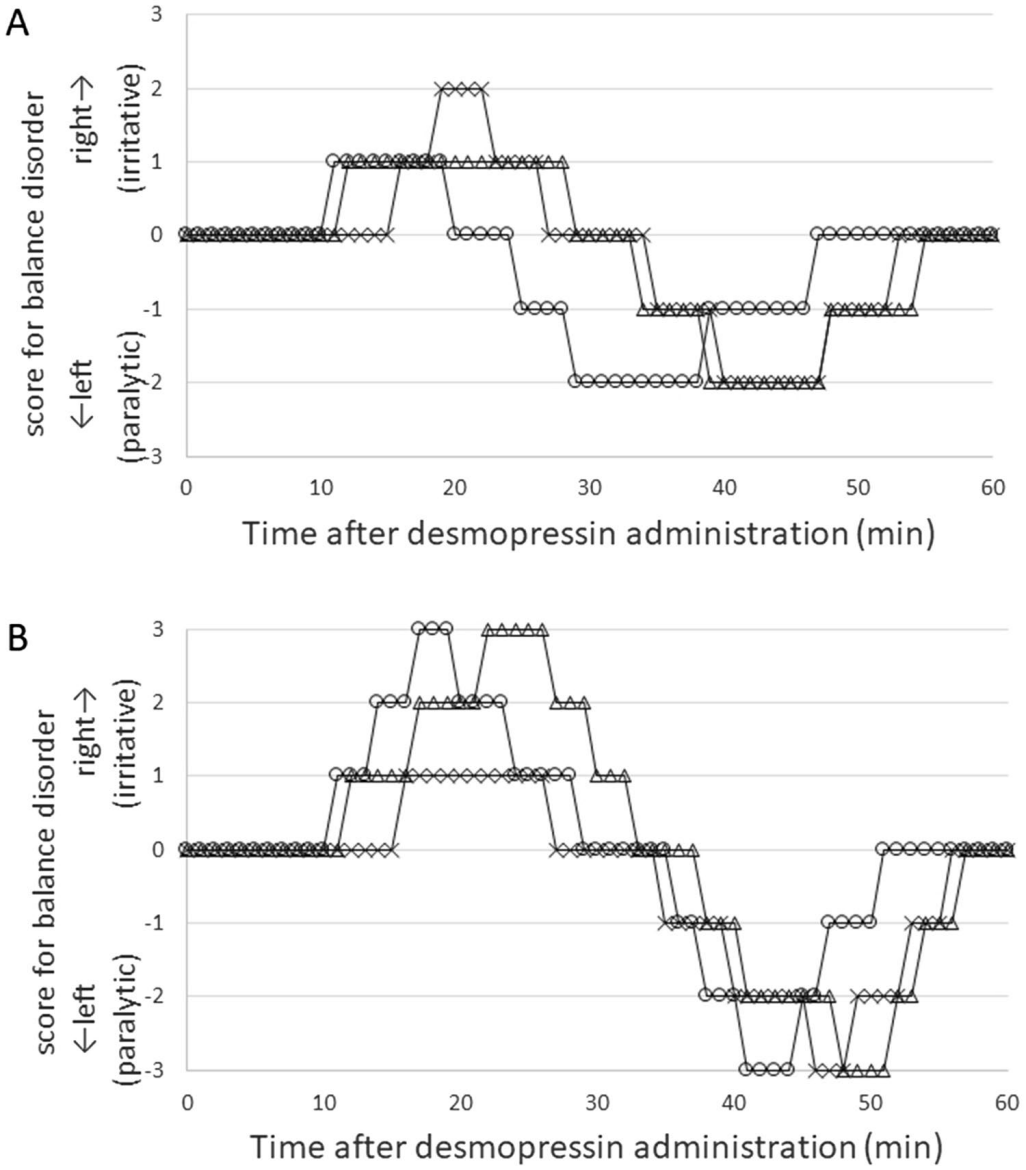
Time course of balance disorder after desmopressin administration. (**A**) Animals administered desmopressin 1 week after electrocauterization of the ES (n = 3). (**B**) Animals administered desmopressin 4 weeks after electrocauterization of the ES (n = 3). Balance disorder, such as falling down and/or circling, were scored from − 3 to 3 as follows: 0, no visible signs; 1 and − 1, slight presence of the irritative and paralytic signs; 2 and − 2, clear evidence of the irritative and paralytic signs; and 3 and − 3, the maximum expression of the irritative and paralytic signs, respectively, according to Ito et al.^31^ We defined irritative and paralytic balance disorders as falling down and circling to the right or left (surgical side), respectively. Each symbol represents 1 animal.

We confirmed our previous results that all animals administered desmopressin 1 or 4 weeks after electrocauterization of the ES developed spontaneous nystagmus (Fig. 5 and Supplementary Movie 2 in Supplementary Materials) and balance disorder (Fig. 6 and Supplementary Movie 3 in Supplementary Materials)^16^. No animal had balance disorder or nystagmus after only surgery. The direction of nystagmus changed from left to right, suggesting a change from irritative to paralytic nystagmus. In general, 10 min after the onset of irritative nystagmus, the direction of nystagmus changed to paralytic; however, there were individual differences. Balance disorder exhibited the same pattern as spontaneous nystagmus with individual differences. In general, 10 min after the onset of irritative balance disorder, the balance disorder changed to paralytic. The duration of balance disorder was approximately 1 h.

### Cochlear function

**Figure 7:**
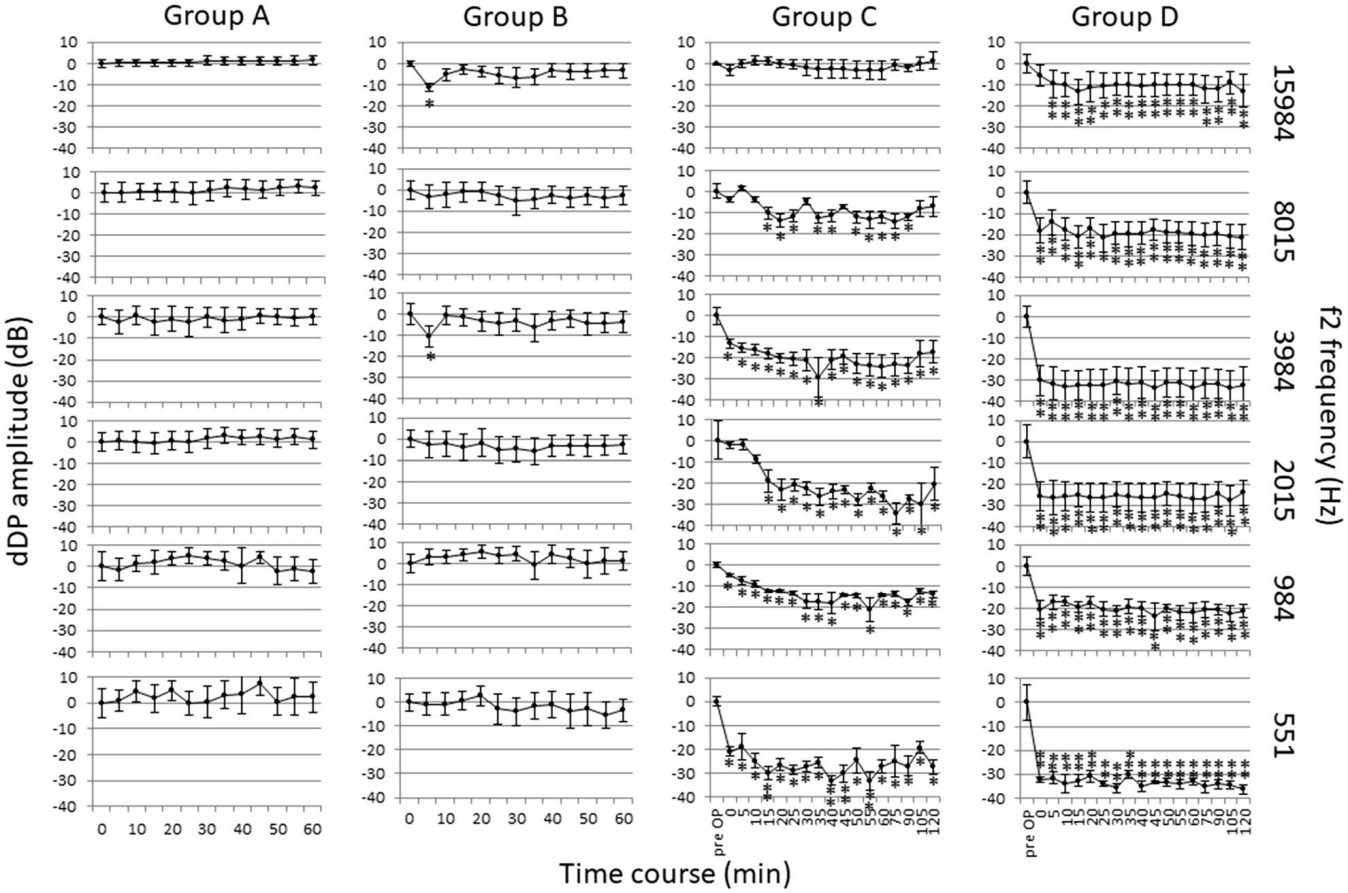
Time course of the amplitude change of DPOAE. Group A: The normal animals underwent DPOAE measurement before and after Ringer’s solution was infused into the jugular vein. The amplitude of DPOAE did not change in any frequency areas after the administration of Ringer’s solution. Group B: The normal animals underwent DPOAE measurement before and after 50 μg/kg of desmopressin was infused into the jugular vein. The amplitude of DPOAE decreased transiently in a limited frequency area with a significant difference after desmopressin administration. Group C: The animals underwent DPOAE measurement before the left ES was obliterated (pre-OP), and before (0 min) and after (5–120 min) 50 μg/kg of desmopressin was infused into the jugular vein 1 week after obliteration. The amplitude of DPOAE decreased significantly for up to 2 h, with slight recovery in the low frequency area. Group D: The animals underwent DPOAE measurement before the left ES was obliterated (pre-OP), and before (0 min) and after (5–120 min) 50 μg/kg of desmopressin was infused into the jugular vein 4 weeks after obliteration. The amplitude of DPOAE decreased significantly 4 weeks after ES ablation, except in the 16-kHz area. In the 16-kHz area, the amplitude of DPOAE decreased after the administration of desmopressin, although the DPOAE decrease was in a wide frequency area. Data are presented as the mean ± SE. Data were compared by Tukey’s test and changes were regarded as significant when *P* < 0.05. *P < 0.05; **P < 0.01.

The time-course of the amplitude change of DPOAE after ablation of the ES and /or administration of desmopressin is shown in Fig. 7. In group A, the amplitude of DPOAE did not change in any frequency areas after the administration of Ringer’s solution in normal animals. This suggests that the recording of DPOEA was stable. In group B, the amplitude of DPOAE decreased transiently in a limited frequency area, with a significant difference after desmopressin administration in normal animals. This suggests that the increase in the VP V2 agonist has minimal effects in normal animals. In group C, the amplitude of DPOAE decreased significantly for up to 2 h, with slight recovery in the lower-frequency area after desmopressin administration in animals 1 week after ES ablation. This suggests that the increase in the VP V2 agonist induced low-tone sensorineural hearing loss in animals with dysfunction of the ES. These animals represented the early stage of MD in which attacks of vertigo continue with variable remission and the possible development of fluctuating low-tone hearing loss. In group D, the amplitude of DPOAE decreased significantly 4 weeks after ES ablation, except in the 16-kHz area. In the 16-kHz area, the amplitude of DPOAE decreased after the administration of desmopressin, although the decrease was in a wide frequency area. This suggests that severe EH induced by keeping animals for 4 weeks after ES ablation caused marked hearing loss.

## Discussion

The present study suggested that EH was aggravated by ES dysfunction combined with the effects of overproduction of endolymph by acute V2 effects due to acute systemic desmopressin administration. The histopathological findings related to ES in patients with MD are due to: poor development of the ES^23,24^, fibrotic changes of the ES and a hypoplastic vestibular aqueduct^25,26^, and acute V2 effects causing marked changes in inner ear homeostasis, leading to vestibular and cochlear attacks. We previously developed a new clinically relevant animal model, which exhibits acute periods of vestibular dysfunction similar to the vestibular attacks in patients with MD^16^. In the current study, we confirmed the previously reported vestibular disorder in an animal model, and observed acute periods of low-tone sensorineural hearing loss in animals that survived for only 1 week after electrocauterization of the left ES and the administration of desmopressin. These animals also developed irritative and/or paralytic nystagmus^16^ (Movie 2). Concerning acute attacks of Meniere's disease, the membrane rupture theory states that attacks develop when endolymph, with its high potassium ion concentration, escapes into the perilymph and surrounds a first-order neuron^27,28^. However, there was no rupture of Reissner’s membrane in our animal model. This suggests that acute attacks of Meniere's disease are caused by the abrupt distention of Reissner’s membrane. Two major hypotheses that have been proposed and support this speculation are excessive endolymphatic pressure followed by leaky membranes and the subsequent mixing of high potassium ion concentration endolymph with perilymph. Experimentally, the distention of Reissner’s membrane causes increases in the potassium ion concentrations in the scalae vestibuli and tympani^29^. These increases in potassium ion concentrations are likely toxic to hair cells and auditory nerve fibers, and may cause acute attacks of Meniere’s disease.

As in our previous report^16^, animals administered desmopressin 1 or 4 weeks after electrocauterization of the ES developed both spontaneous nystagmus and balance disorder. In the current study, the animals kept for 4 weeks exhibited marked sensorineural hearing loss and a decrease in the amplitude of DPOAE after the administration of desmopressin in the 16-kHz area only. This suggests that the remaining hearing will also be aggravated by VP even though hearing loss was noted in a wide frequency area.

In this study, we examined the vestibular and cochlear function, performed live imaging of the cochlea in our animal model of MD, and discussed possible causes of acute attacks in MD. However, many possible etiological factors lead to hydrops, which in turn causes clinical symptoms^30^. We only clarified one aspect of MD in this study and further studies are needed to understand the entire picture of MD.

## Materials and methods

### Surgical procedure for electrocauterization of the endolymphatic sac

The animals were anesthetized by intramuscular injection of ketamine (35 mg/kg) and xylazine (5 mg/kg). They were placed in a prone position with a head holder and operated on under sterile conditions. A dorsal midline scalp incision was made under local anesthesia with xylocaine. The left occipital bone was removed to expose the endolymphatic sac via an epidural occipital approach. We drilled around the temporo-occipital suture to the skeletonized sigmoid sinus and retracted it medially to reveal the operculum. After visualization of the endolymphatic sac, the extraosseous portion of the sac was cauterized electrically in order to not injure the sigmoid sinus with the bipolar electrocoagulator (Surgitron Model FFPF; Ellman International Inc., Hewlett, NY, USA). The operation was performed using an Olympus operating microscope. As we did not drill the temporal bone, there was no risk to damage of the posterior semicircular canal. In addition, our previous morphological examination of the temporal bone after this surgery revealed no damage of the posterior semicircular canal (Supplementary Fig. 2 in Supplementary Materials).

### In vivo OCT imaging

**Figure 8:**
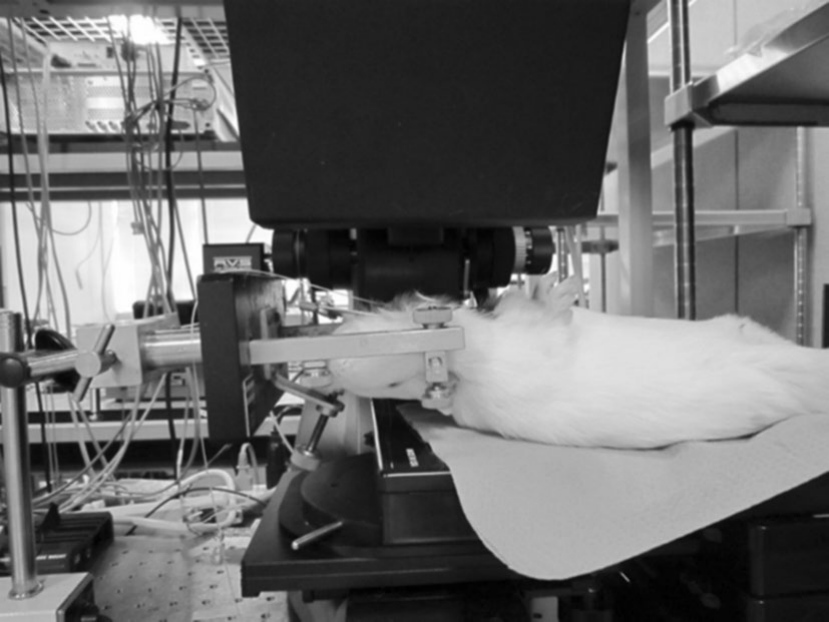
Setting for in vivo OCT imaging. A guinea pig was mounted on the stage with a head holder after the bulla was opened via a ventral approach.

We obtained mid-modiolar section images of the cochleae using the Santec OCT system (Santec Co., Aichi, Japan) as follows: The characteristics of the Santec OCT system were as follows: The center wavelength band was 1,320 nm and the band width was 90 nm. The axial and lateral resolutions were 12.0 and 17.0 µm, respectively. The measurement speed and frame rate were 50,000 lines/scan and 100 frames/s, respectively. The image depth and width were 6.0 and 10.0 mm, respectively. The animals were anesthetized by intramuscular injection of ketamine (35 mg/kg) and xylazine (5 mg/kg). A guinea pig was mounted on the stage with a head holder (SG-1; Narishige, Japan) after the bulla was opened via a ventral approach (Fig. 8). Fifteen Hartley guinea pigs with a positive Preyer’s reflex weighing approximately 300 g were used, and were divided into the normal, hydrops, and hydrops with desmopressin groups, with five animals in each group. In the normal group, the internal structures of the cochlea were observed in vivo as a control. In the hydrops group, the animals underwent electrocauterization of the left endolymphatic sac. Four weeks later, we observed the internal structures of the cochlea in vivo. In the hydrops with desmopressin group, the animals underwent electrocauterization of the left endolymphatic sac. One week later, 50 μg/kg of desmopressin was infused into the jugular vein and we observed the internal structures of the cochlea in vivo.

**Figure 9:**
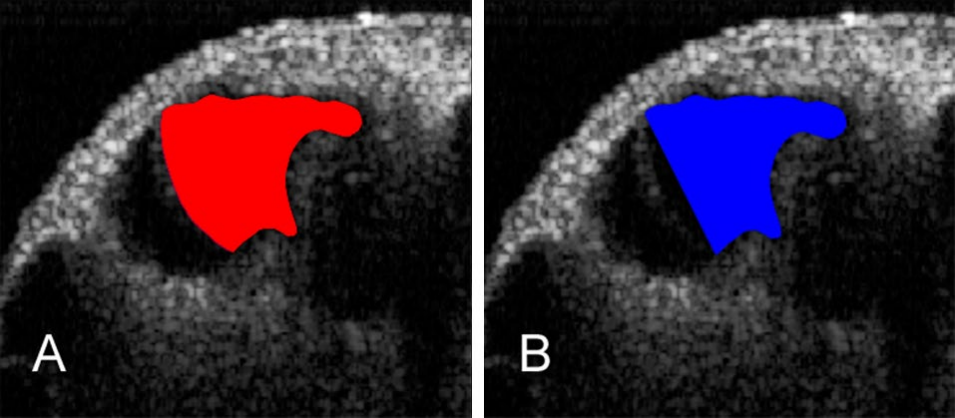
Parameters for quantitative assessment of the changes in the endolymphatic space, organ of Corti, and lateral wall. (**A**) The cross-sectional area of the dilated scala media (red area) in an OCT image. (**B**) The cross-sectional area of the scala media (blue area) is enclosed by a straight line segment. This line segment represents the position of the predicted Reissner’s membrane at the upper margin of the stria vascularis to its normal medial attachment at the spiral limbus.

For quantitative assessment of the endolymphatic space, we used the digital image measurement software, Micro Analyzer Ver 1.1 (Nippon Poladigital Co. Ltd, Tokyo, Japan). For quantitative assessment of the endolymphatic space and variations of the cochlea, we measured the increase in the cross-sectional area of the scala media (IR) compared with that in the mid-modiolar sections. For this analysis, we used the following two parameters in the apical turns: (1) the cross-sectional area of the dilated scala media (Fig. 9 A: red area) and (2) the cross-sectional area of the original scala media (Fig. 9 B: blue area), which was enclosed by a straight line segment. This line segment represents the position of the predicted Reissner’s membrane at the upper margin of the stria vascularis to its normal medial attachment at the spiral limbus. From these parameters, we calculated the increase (%) in the cross-sectional area of the scala media (increasing ratio: IR) in the apical turn using the following formula: IR (%) = 100 × (red area − blue area)/blue area.

Data were compared by Tukey’s test and changes were regarded as significant when *P* < 0.05. All the changes and differences mentioned in the text were significant.

### Vestibular function

In total, 12 pigmented guinea pigs with a positive Preyer’s reflex weighing approximately 300 g were used. All animals underwent electrocauterization of the ES in the left ear and were maintained for 1 or 4 weeks before the administration of desmopressin. Three animals in each group were assessed for signs of balance disorder and the presence of nystagmus for 1 h after they received a subcutaneous injection of 100 μg/kg (25 mL/kg) of desmopressin acetate hydrate. We recorded spontaneous nystagmus using an eye movement recording system in a dark room for 1 h after the administration of desmopressin. The recording procedure was described previously^16^. Briefly, a guinea pig was mounted on the table with a headholder (SG-1; Narishige, Japan). The head was tilted nose-down such that the lateral semicircular canals were positioned approximately parallel to the horizontal plane. The table was surrounded by a blackout curtain. To monitor eye movements, the right eye was illuminated by an infrared LED (TLN201, Toshiba, Japan) and monitored by a small infrared-sensitive CCD camera (C53500; Tokyo Electronic Industry, Japan). The LED and camera were both fixed to the table. The number of nystagmus was counted manually.

In the open field, we monitored posture for one hour after the administration of desmopressin to assess the presence of balance disorder such as falling down and/or circling. Circling represents a stereotyped rotatory movement in which the animal circles around itself. We defined irritative and paralytic balance disorders as falling down and circling to the right or left (surgical side), respectively. These behaviors were scored from − 3 to 3 as follows: 0, no visible signs; 1 and − 1, slight presence of the irritative and paralytic signs; 2 and − 2, clear evidence of the irritative and paralytic signs; and 3 and − 3, the maximum expression of the irritative and paralytic signs, respectively, according to Ito et al.^31^ Postural disturbances during a vestibular attack were recorded.

### Cochlear function

Twenty-two Hartley guinea pigs with a positive Preyer’s reflex weighing approximately 300 g were used and divided into four groups. They underwent DPOAE measurement with an acoustic probe using the DP2000 DPOAE measurement system version 3.0 (Starkey Laboratory, Eden Prairie, MN, USA). In group A (n = 5), the animals underwent DPOAE measurement before and after Ringer’s solution was infused into the jugular vein as a control. In group B (n = 7), the animals underwent DPOAE measurement before and after 50 μg/kg of desmopressin was infused into the jugular vein. In group C (n = 5), the animals underwent DPOAE measurement before the left ES was obliterated, and before and after 50 μg/kg of desmopressin was infused into the jugular vein 1 week after obliteration. In group D (n = 5), the animals underwent DPOAE measurement before the left ES was obliterated, and before and after 50 μg/kg of desmopressin was infused into the jugular vein 4 weeks after obliteration. DP-grams comprised 2f(1)–f(2) DPOAE amplitudes as a function of f2. Acoustic stimuli were as follows: f(1) 65 dBSPL, f(2) 55 dBSPL, f(2)/f(1) = 1.22, f(2) 0.5–16 kHz. Data reflecting changes in DPOAE amplitudes are presented as the mean ± SE. Data were compared by Tukey’s test and changes were regarded as significant when *P* < 0.05. All the changes and differences mentioned in the text were significant.

These experiments were approved by the Tokyo University Animal Care and Use Committee (#H12-151), and were conducted in accordance with The Animal Welfare Act and the guiding principles for animal care set by the Ministry of Education, Culture, Sports and Technology, Japan.

## Supplementary information

Supplementary Movie 1.

Supplementary Movie 2.

Supplementary Movie 3.

Supplementary Figure 1.

Supplementary Figure 2.
