# Supplementary figures and images for: Live imaging and functional changes of the inner ear in an animal model of Meniere’s disease
Source: Sci Rep. 2020 Jul 23;10:12271. doi: 10.1038/s41598-020-68352-0 (PMC7378199; doi:10.1038/s41598-020-68352-0)

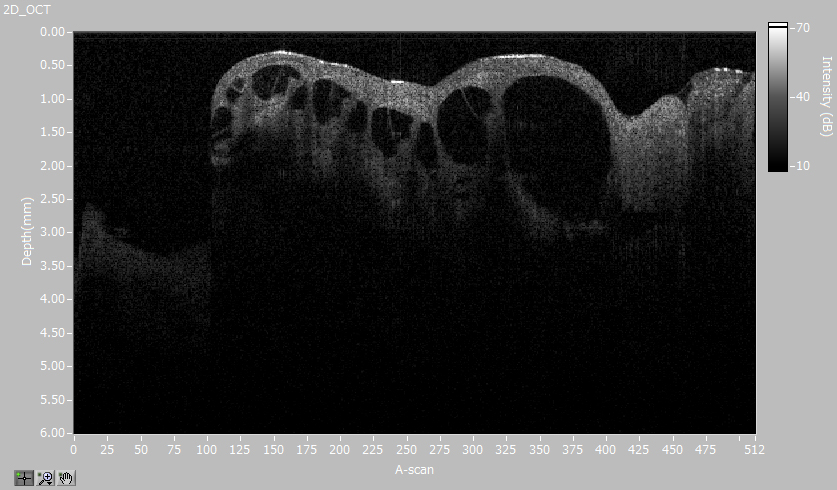

Supplement: Supplementary file 4 — Supplementary Figure 1. [file 41598_2020_68352_MOESM4_ESM.jpg]

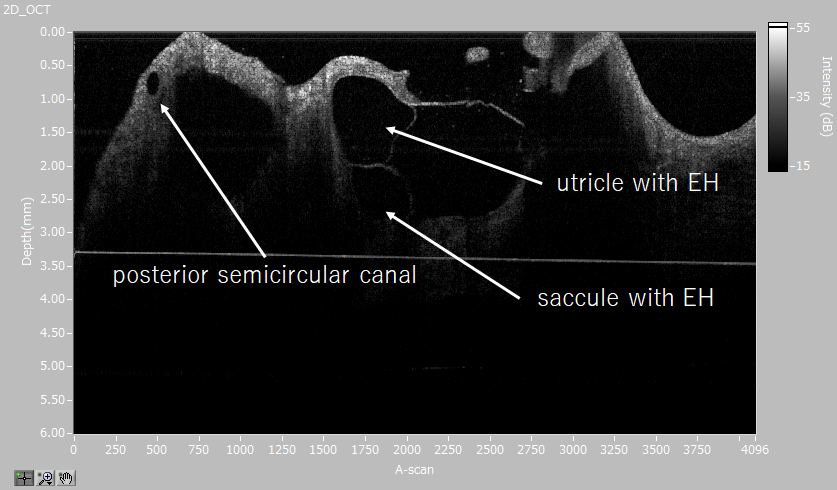

Supplement: Supplementary file 5 — Supplementary Figure 2. [file 41598_2020_68352_MOESM5_ESM.jpg]
